# A brief overview of targeted radionuclide therapy trials in 2022

**DOI:** 10.3389/fnume.2023.1169650

**Published:** 2023-06-23

**Authors:** Aidan Healy, Elaine Ho, Phillip Kuo, Katherine Zukotynski

**Affiliations:** ^1^ California Northstate University College of Medicine, California Northstate University, Sacramento, CA, United States; ^2^ Michael G. DeGroote School of Medicine, McMaster University, Hamilton, ON, Canada; ^3^ Department of Radiology, University of Arizona, Tucson, AZ, United States; ^4^ Departments of Radiology and Medicine, McMaster University, Hamilton, ON, Canada

**Keywords:** targeted radionuclide therapy, clinical trials, somatostatin receptors, prostate specific membrane antigen, nuclear medicine

## Abstract

There is a growing use of radionuclide therapy for the medical care of oncology patients, where radioactive pharmaceuticals are used to target and treat various cancer types. This paper provides a brief overview illustrating the spectrum of ongoing and recently completed radionuclide therapy clinical trials in oncology. The trials selected highlight the potential of radionuclide therapies to provide a promising treatment option across a spectrum of cancer patients, while also discussing the importance of patient selection and monitoring, as well as potential side effects and safety concerns. Ultimately, the results of these trials will be crucial in determining the future use of radionuclide therapies in cancer treatment.

## 1. Introduction

The field of nuclear medicine is ever expanding to include an increasing array of targeted radionuclide therapies (TRTs) capable of treating oncology patients. Although TRT has existed for many years, recently it is gaining attention due to the potential for prolonging patient survival across differing cancer types, often with minimal toxicity. While thyroid cancer, neuroendocrine tumors and prostate cancer remain the most common targeted cancer types, additional malignancies continue to be added to the list. In this paper we provide a short summary of the current spectrum of clinical trials using TRT based on an analysis of those studies listed on clinicaltrials.gov (1). The studies chosen were based on a search of clinicaltrials.gov up to and including February 14, 2023, for all ongoing and recently completed clinical trials and then selecting those trials that included TRTs across a spectrum of disease. These studies are categorized by the targeted receptor, treatment indication, and clinical trial phase. We also briefly discuss obstacles for TRT research, current gaps in this research and future developments. Trials are summarized in Table 1.

**Table 1:** Summary of active or recently completed studies illustrating a Spectrum of radiopharmaceutical advances.

Receptor	Radionuclide	Study drug	Conditions	Accrual goal	Status/date of completion	Phase	NCT
SSTR	^90^Y	^90^Y-DOTA-tyr3-Octreotide	Neuroendocrine Tumor, Carcinoid Tumor, Neuroblastoma, Medulloblastoma	39	5/27/20^a^	2	NCT03273712
	^67^Cu	^67^Cu-SARTATE	Neuroblastoma, Relapsed Neuroblastoma and Refractory Neuroblastoma	34	Recruiting	2	NCT04023331
	^68^Ga	^68^Ga-DOTATATE-	Hepatocellular Carcinoma	12	2/3/22^b^	2	NCT03648073
PSMA	^177^Lu	^177^Lu-PSMA-617	Progressive Metastatic Castration Resistant Prostate cancer	200	12/31/21^c^	2	NCT03392428
	^177^Lu	^177^Lu-DOTA-TLX591	Biochemically Recurrent Oligometastatic, Prostate Specific Membrane Antigen-Expressing Prostate Cancer	50	Recruiting	2	NCT05146973
	^67^Cu	^67^Cu-SAR-bisPSMA	Metastatic Castrate Resistant Prostate Cancer (mCRPC)	44	Recruiting	2	NCT04868604
	^177^Lu	^177^Lu-PSMA-617	Prostatic Neoplasms	20	Active/Not Recruiting	2	NCT04430192
	^177^Lu	^177^Lu-ITG-PSMA-1	Soft Tissue Sarcoma	20	Recruiting	1	NCT05420727
FAP	^177^Lu	^177^Lu-DOTA-FAPI	Locally Advanced or Metastatic Cancer	30	Recruiting	1	NCT04849247
	^177^Lu	^177^Lu-FAP-2286	Advanced Solid Tumors	170	Recruiting	1 + 2	NCT04939610
FGFR3	^225^Ac	^225^Ac-FPI-1966, ^111^In-FPI-1967	Advanced Solid Tumor, Head and Neck Squamous Cell Carcinoma, Bladder Carcinoma, Susceptible FGFR3 Genetic Alterations, FGFR3, FGFR3 Protein Overexpression, Ovarian Cancer, Colorectal Cancer, Breast Cancer, Liver Cancer, Lung Cancer, Gastric Cancer	155	Recruiting	1 + 2	NCT05363605
CCK2 Receptor—Gastrin analog	^177^Lu	^177^Lu-PP-F11N	Thyroid Cancer, Medullary; Neuroendocrine Tumor of the Lung Grade 1 and 2; Neuroendocrine Tumor of the Thymus Grade 1 and 2; Neuroendocrine Tumor GEP Grade 1-3	24	Recruiting	1	NCT02088645
CD46	^89^Zr	^89^Zr-DFO-YS5	mCRPC	24	Recruiting	1	NCT05245006

^a^ Results posted 2/9/2023.

^b^ Results posted 1/10/23.

^c^ Updated 6/13/22.

Perhaps one of the most significant obstacles for recent clinical trials including TRT, was the COVID-19 pandemic, that caused many of the studies listed on clinicaltrials.gov to be paused or pre-maturely “completed” because of the associated strain on healthcare resources and shelter-in-place policies that resulted in disruptions to clinical trial activities. Another obstacle inherent in clinical trials including TRT is radiation exposure. While TRTs are designed to target and destroy specific types of cancer cells, they can also damage healthy tissues, leading to adverse effects. One way to reduce the risk of radiation exposure is through pre-therapy imaging and then using this as a tool to optimize the amount of radiopharmaceutical given to provide optimal therapy while minimizing toxicity through exposure to surrounding healthy tissues. In addition, technical advances have enabled the development of increasingly targeted radionuclides with lower off-target exposure. Today, common side-effects associated with TRTs include fatigue, nausea and bone marrow suppression; however, nephrotoxicity, among other side-effects may also be seen (2, 3). In certain instances, the co-administration of agents to help reduce side effects and toxicity are given. Example of ongoing trials that have adopted this approach include a Phase 1 trial using ^111^In-CP04 to target the CCK2 receptor that includes the co-administration of gelofusine/gelaspan, colloidal plasma substitutes, to minimize nephrotoxic effects and myelosuppression. Another example is a Phase 2 trial using ^68^Ga-DOTATOC and ^90^Y-DOTATOC that includes the co-administration of lysine and arginine for nephroprotection. Also, in many trials a diagnostic radiopharmaceutical is used to image patients prior to TRT in order to detect patients most likely to benefit and exclude those most likely to experience adverse effects. For example, in patients with hepatocellular carcinoma (HCC), ^68^Ga-DOTATATE-PET is done first and only those patients with adequate somatostatin receptor (SSTR) expression on imaging to suggest benefit of the TRT are given ^177^Lu-DOTATATE. Another trial includes a diagnostic scan with ^68^Ga-DOTA-5G for patients with pancreatic cancer, where only patients with adequate uptake (defined as SUVmax >2-fold above normal lung or liver) are given the TRT, specifically ^177^Lu-DOTA-ABM-5G. In another trial ^203^Pb-VMT01 and ^68^Ga-VMT02 are used for image screening to detect patients with melanoma likely to benefit from ^212^Pb-VMT01.

An ongoing and important gap in research involving TRTs is the lack of large-scale clinical trials capable of providing high-quality evidence of the safety and efficacy of these treatments. While there have been many small studies and case reports suggesting promising results and a few larger trials that have impacted clinical practice, there is a need for more large, well-designed clinical trials to validate early phase clinical trial findings and ultimately inform/change clinical practice on a broad scale (4). In addition, there is a need for further research on radiotracers with improved pharmacokinetic and biodistribution profiles, as well as greater specificity for cancer subtypes. This requires understanding the biology underlying different cancer subtypes, as well as continued advancement in radiopharmaceuticals and imaging techniques to ensure precise targeting of the radiopharmaceutical to the tumor. Another gap in research is determining the best therapy combination to improve overall efficacy and tolerability. For example, cryoablation of disease sites or and/or immunotherapy, among others may have a synergistic effect with TRT.

Finally, most current trials target SSTR and prostate specific membrane antigen (PSMA) receptors with innovations focusing on the development of radiopharmaceuticals that more accurately target these receptors, or that apply already existing TRT to new cancer subtypes. For example, a feasibility study was recently conducted to determine if patients with HCC expressed SSTR adequately to benefit from ^177^Lu-DOTATATE treatment. Future developments in the field may increasingly focus on identifying new receptors that can serve as TRT targets; currently, many trials focusing on this are early in the development process. For example, there is a handful of phase 1 trials targeting the fibroblast growth factor receptor 3 (FGFR3), fibroblast activation protein inhibitor (FAPI), cholecystokinin 2 receptor (CCK2), melanocortin sub-type 1 receptor (MC1R) and the chemokine receptor 4 (CXCR4), among others. There are no trials at this time that target the gastrin-releasing peptide receptor (GRP-R) or the integrin αVβ3 or αVβ5 receptors, which have also been identified as potential targets for neuroendocrine tumors (5). Investigating new receptors has the potential to extend TRT to a variety of cancers subtypes, and could improve the specificity of TRT, which continues to be a barrier for effectiveness.

## 2. Examples of clinical trials targeting SSTR

Somatostatin receptors are overexpressed in a variety of neuroendocrine tumors (NETs), making them attractive targets for radionuclide therapy. Radiolabeled somatostatin analogs such as octreotide and DOTATATE have been developed to target SSTRs for imaging and therapy by delivering targeted radiation directly to tumor cells. The use of radiolabeled somatostatin analogs for therapy has shown promise in clinical trials. Specifically, a phase 3 clinical trial showed that ^177^Lu-DOTATATE improved progression-free survival in patients with midgut NETs compared to standard therapy (6). A phase 3 clinical trial found that ^177^Lu-DOTATOC, a radiolabeled somatostatin analog, improved progression-free survival in patients with gastroenteropancreatic NETs (7). In addition, ^177^Lu and ^90^Y-labeled PRRT have been used in clinical trials showing promise in treating gastroenteropancreatic and bronchial NETs (2). Research is ongoing in an effort to optimize somatostatin receptor-targeted therapy, including determining the optimal amount of TRT to be effective across a spectrum of patient populations, selecting patients most likely to benefit and using existing TRTs in new cancer subtypes. A few examples illustrating the spectrum of ongoing clinical trials is given below.

**NCT03273712** is a trial designed to evaluate the safety and efficacy of Dosimetry-Guided, Peptide Receptor Radiotherapy (PRRT) with ^90^Y-DOTA-tyr3-Octreotide (^90^Y-DOTATOC) in patients with inoperable, somatostatin receptor-positive neuroendocrine tumors. This trial will use dosimetry to determine an individualized radiation dose for each patient, with a maximum of 4 treatment cycles given at 8–12 week intervals (8).

**NCT04023331** aims to evaluate the safety and efficacy of ^67^Cu-SARTATE in pediatric patients with high-risk neuroblastoma through an adaptive and personalized design. The trial consists of two phases, a dose escalation phase, and a cohort expansion phase. Dose escalation will use a modified 3 + 3 study design with up to 4 cohorts of increasing doses monitoring pre-defined Dose Limiting Toxicities for 6 weeks post administration of one therapy cycle of ^67^Cu-SARTATE. Patients who benefit may be offered additional therapy cycles, up to a maximum of 4. Once the Maximum Tolerated Dose (MTD) is established, or Cohort 4 is completed, the study will be expanded to enroll an additional 10 subjects who will receive at least 2 therapy cycles of ^67^Cu-SARTATE at the MTD dose level (9).

**NCT03648073** aims to use ^68^Ga-DOTATATE to determine HCCs expressing SSTR levels deemed sufficiently high to benefit from targeted radionuclide therapy with ^177^Lu-DOTATATE. If successful, this approach may offer a new therapeutic option for HCC patients who are not candidates for other therapies (10).

## 3. Example of clinical trials targeting PSMA

Prostate-specific membrane antigen (PSMA) is a type II transmembrane glycoprotein that is highly expressed in prostate cancer cells. PSMA has been the focus of much research in recent years as a target for radionuclide therapy in men with prostate cancer. In the case of PSMA based TRT, the radioactive isotopes are attached to PSMA-targeting molecules that bind to PSMA on the surface of prostate cancer cells. One of the most ubiquitous radionuclides for PSMA-targeted therapy is ^177^Lu. Clinical trials have shown that ^177^Lu-PSMA therapy improves progression-free survival and overall survival in men with metastatic castration resistant prostate cancer (mCRPC) who have failed other therapies (11, 12) and this TRT was approved for use by the FDA in 2022. Other radionuclides, such as ^225^Ac and ^227^Th, are currently being investigated for PSMA-targeted therapy, with promising preclinical results (13). The examples below illustrate the spectrum of ongoing trials focusing on expanding earlier promising results of TRT in men prostate cancer, assessing combination therapy with TRT, assaying novel TRT agents, evaluating where in the spectrum of disease TRT has the best effect and assaying known TRT agents in novel cancer types.

**NCT03392428** aims to evaluate the efficacy of ^177^Lu-PSMA-617 in men with metastatic prostate cancer who have progressed despite hormonal therapy and chemotherapy. The trial compares the effects of ^177^Lu-PSMA radionuclide therapy with cabazitaxel chemotherapy (14). This study will recruit 200 participants from sites across Australia to expand on the previous randomized phase 2 trial (TheraP) that showed ^177^Lu-PSMA-617 led to a higher PSA response and fewer grade 3 or 4 adverse events compared with cabazitaxel published in 2022, suggesting TRT could be a good alternative to cabazitaxel (15).

**NCT05146973** aims to assess the effectiveness of ^177^Lu-TLX591, a radiolabelled PSMA-targeting antibody, in combination with external beam radiation therapy (EBRT) for the treatment of biochemically recurrent, oligometastatic, PSMA-expressing prostate cancer. The therapeutic potential of TLX591 lies in its ability to target PSMA-expressing tumors by radiolabeling it with a therapeutic radioactive isotope (16).

**NCT04868604** is a phase 1/2 clinical trial that aims to evaluate the safety, dosimetry, and therapeutic potential of two copper-labeled PSMA-targeting agents, ^64^Cu-SAR-bisPSMA and ^67^Cu-SAR-bisPSMA, for identifying and treating PSMA-expressing metastatic castration-resistant prostate cancer (17).

**NCT04430192** assesses the safety, effectiveness, and appropriate dosage of ^177^Lu-PSMA in men with high PSMA-expressing high-risk localized or locoregional advanced prostate cancer undergoing radical prostatectomy and pelvic lymph node dissection. The trial evaluates the radiation dose absorbed, imaging response, biochemical response, pathological response, adverse effects, surgical safety, and quality of life. Patients will receive one or two cycles of ^177^Lu-PSMA before surgery (18).

**NCT05420727** evaluates the use of ^68^Ga-PSMA-11 PET/CT for imaging and ^177^Lu-ITG-PSMA-1 treatment, in patients with soft tissue sarcomas (19).

## 4. Examples of clinical trials targeting novel receptors

There are early phase trials targeting a host of receptors. For example, there are several types of Fibroblast Growth Factor Receptors (FGFR), transmembrane receptors associated with cell growth and invasiveness. Mutations in this receptor have been associated with a spectrum of tumor types. Radioimmunotherapy (RIT) is a type of targeted therapy that involves using a radioactive isotope conjugated to a monoclonal antibody (mAb) to selectively deliver radiation and immunotherapy to cancer cells expressing the targeted protein. Studies have suggested that FGFR3 is associated with bladder cancer and multiple myeloma (20). Research is ongoing to optimize the clinical application, dosing schedule and combination of therapies including FGFR3-targeted therapy. **NCT05363605** is an example of an early phase trial evaluating safety, tolerability, and distribution of ^225^Ac-FPI-1966, ^111^In-FPI-1967, and vofatamab (anti-FGFR3 antibody) in patients with FGFR3-expressing solid tumors. The study includes 5 dose escalation cohorts, and a subsequent expansion cohort of two tumor-specific cohorts and one basket cohort. The study evaluate the impact of vofatamab given prior to TRT on the dosimetry and tolerability of ^225^Ac-FPI-1966 and ^111^In-FPI-1967 (21).

Several tumor types are characterized by a strong desmoplastic reaction resulting in cancer-associated fibrosis and many of these fibroblasts differ from normal fibroblasts through their expression of a Fibroblast Activation Protein (FAP). **NCT04849247** is an early phase trial investigating the safety and efficacy of ^68^Ga-DOTA-FAPI and ^177^Lu-DOTA-FAPI, to diagnose and treat a spectrum of advanced or metastatic cancers. A baseline PET/CT with ^68^Ga-DOTA-FAPI is used to identify patients eligible for ^177^Lu-DOTA-FAPI therapy, which is then administered in escalating doses to determine the Recommended Phase 2 Dose (RP2D) of the treatment (22). **NCT04939610** is a similar study that investigates the safety and efficacy of ^68^Ga-DOTA-FAPI and ^177^Lu-DOTA-FAPI in patients with locally advanced or metastatic cancer. This study also uses a ^68^Ga-DOTA-FAPI PET/CT scan to identify patients eligible for ^177^Lu-DOTA-FAPI therapy, which is administered in escalating doses to obtain the RP2D of ^177^Lu-DOTA-FAPI (23).

CCK2 receptors have been shown to be overexpressed in various cancers, including medullary thyroid cancer (MTC). Radiolabeled peptides, such as ^177^Lu-DOTA-CCK, have been assayed in the treatment of MTC (24). Also, use of ^177^Lu-DOTA-CCK in combination with other treatments, such as chemotherapy, may have promise in improving overall survival rates in patients with advanced MTC. **NCT02088645** aims to investigate ^177^Lu-PP-F11N, a gastrin analog, for imaging and therapy in patients with advanced medullary thyroid carcinoma (MTC), as well as gastroenteropancreatic-neuroendocrine tumors (GEP-NET) and NETs of the lung or thymus. The trial consists of two phases: a pilot study to evaluate tumor detection and a dose escalation phase to determine the maximum tolerated dose of ^177^Lu-PP-F11N in patients with MTC. The study will also assess the correlation between dose and treatment response, as well as organ radiation exposure and the maximum tolerated dose, in order to develop individualized therapy planning (25).

CD46 is a membrane-bound complement regulator protein that is overexpressed in various cancer cells. CD46-targeted radionuclide therapy has shown promising results in preclinical studies, with significant tumor growth inhibition observed in various tumor models (26). **NCT05245006** is an early phase study that aims to investigate the feasibility and safety of targeting CD46 in mCRPC using an imaging biomarker and an antibody-drug conjugate (27).

Melanocortin sub-type 1 receptor (MC1R) is a G protein-coupled receptor that plays a critical role in skin pigmentation and is involved in the regulation of cell proliferation and differentiation. MC1R has been identified as a potential target for radionuclide therapy in melanoma, as its expression is highly upregulated in melanoma cells compared to normal skin cells. Preclinical studies have investigated the use of radiolabeled alpha-melanocyte-stimulating hormone (α-MSH), a natural ligand of MC1R, for targeted radionuclide therapy of melanoma. **NCT04904120** aims to evaluate the safety and feasibility of using the agents (^203^Pb-VMT01 and ^68^Ga-VMT02) to image melanoma tumors expressing the melanocortin sub-type 1 receptor (MC1R). The study has a cross-over design in which participants with stage IV or inoperable stage III metastatic melanoma serve as their own comparators. The primary outcome measure is the safety and tolerability of the imaging agents, and the results will be used to guide the development of imaging and dosing for future trials of ^212^Pb-VMT01 in the treatment of metastatic melanoma (28).

## 5. Conclusion

The field of nuclear medicine is growing field and the use of TRT for imaging and therapy is becoming increasingly ubiquitous in clinical trials and routine clinical practice. Obstacle include the risk of radiation exposure and associated adverse events. Often side effects from these therapies include fatigue, nausea and myelosuppression. Several ongoing trials aim to reduce toxicity and enhance efficacy by combining TRT with pre-medication or other therapies. Larger clinical trials are needed to provide high-quality evidence on optimal dosing and timing of when to include TRT in the algorithm of patient care along the course of their disease. A handful of early phase trials illustrate the continued search for novel TRT with improved specificity for cancer cells and identification of new targets beyond SSTR and PSMA.

We hope this brief overview inspires interest in the area of TRT and showcases the current state of radionuclide therapies in clinical trials, and emerging field with very promising new treatment options for our cancer patients.
